# Increased Bioavailability of β-Alanine by a Novel Controlled-Release Powder Blend Compared to a Slow-Release Tablet

**DOI:** 10.3390/pharmaceutics13091517

**Published:** 2021-09-19

**Authors:** Lydia de Salazar, Ignacio Segarra, Francisco Javier López-Román, Antonio Torregrosa-García, Silvia Pérez-Piñero, Vicente Ávila-Gandía

**Affiliations:** 1 Sports Physiology Department, Faculty of Health Sciences, UCAM Universidad Católica San Antonio de Murcia, 30107 Guadalupe, Spain; dssflydia@ucam.edu (L.d.S.); sperez2@ucam.edu (S.P.-P.); vavila@ucam.edu (V.Á.-G.); 2 Department of Pharmacy, Faculty of Health Sciences, UCAM Universidad Católica San Antonio de Murcia, 30107 Guadalupe, Spain; isegarra@ucam.edu; 3 Pharmacokinetics, Patient Care and Translational Bioethics Research Group, UCAM Universidad Católica San Antonio de Murcia, 30107 Guadalupe, Spain; 4 Health Sciences Department, UCAM Universidad Católica San Antonio de Murcia, 30107 Guadalupe, Spain; jlroman@ucam.edu; 5 Biomedical Research Institute of Murcia (IMIB-Arrixaca), 30120 Murcia, Spain; 6 Health Sciences PhD Program, Campus de los Jerónimos N° 135, UCAM Universidad Católica San Antonio de Murcia, 30107 Guadalupe, Murcia, Spain

**Keywords:** β-alanine, pharmacokinetics, paresthesia, bioavailability

## Abstract

Background: β-Alanine is a sport supplement with increasing popularity due to its consistent ability to improve physical performance, with the downside of requiring several weeks of supplementation as imposed to the maximum daily and single dose tolerated without side effects (i.e., paresthesia). To date, the only alternative to overcome this problem has been use of a sustained-release tablet, while powders are the most commonly used format to deliver several grams of amino acids in a single dose. In this study we assessed the bioavailability, pharmacokinetics and paresthesia effect of β-alanine after administration in a novel controlled-released powder blend (test) versus a sustained-release tablet (reference). Methods: Twelve subjects (25.6 ± 3.2 y, 50% female) participated in a randomized, single-blind, crossover study. Each participant was administered orally the test (β-alanine 8 g, l-histidine 300 mg, carnosine 100 mg) or the reference product (10 tablets to reach β-alanine 8 g, Zinc 20 mg) with a 1-week washout period. β-Alanine plasma concentrations (0–8 h) were determined by LC-MS/MS and model-independent pharmacokinetic analysis was carried out. Paresthesia intensity was evaluated using a Visual Analog Score (VAS) and the categorical Intensity Sensory Score (ISS). Results: The C_MAX_ and AUC_0__→__∞_ increased 1.6- and 2.1-fold (both *p* < 0.001) in the test product, respectively, which yielded 2.1-fold higher bioavailability; K_a_ decreased in the test (0.0199 ± 0.0107 min^−1^) versus the reference (0.0299 ± 0.0121 min^−1^) product (*p* = 0.0834) as well as V/F and Cl/F (both *p* < 0.001); MRT_0__→last_ increased in the test (143 ± 19 min) versus reference (128 ± 16 min) formulation (*p* = 0.0449); t_1/2_ remained similar (test: 63.5 ± 8.7 min, reference: 68.9 ± 9.8 min). Paresthesia E_MAX_ increased 1.7-fold using the VAS (*p* = 0.086) and the ISS (*p* = 0.009). AUEC increased 1.9-fold with the VAS (*p* = 0.107) and the ISS (*p* = 0.019) reflecting scale intrinsic differences. Pharmacokinetic-pharmacodynamic analysis showed a clockwise hysteresis loop without prediction ability between C_MAX_, AUC_0__→__∞_ and E_MAX_ or AUEC. No side effects were reported (except paresthesia). Conclusions: The novel controlled-release powder blend shows 100% higher bioavailability of β-alanine, opening a new paradigm that shifts from chronic to short or mid-term supplementation strategies to increase carnosine stores in sports nutrition.

## 1. Background

β-Alanine is a sports supplement with increasing relevance due to its ability to improve physical performance in several mixed sports and exercise tests by trained and untrained individuals [1,2,3,4,5,6,7]. It increases the muscle stores of the intracellular dipeptide carnosine [1,8,9], and it is the rate limiting amino acid in its synthesis when binded with l-histidine. It contains an imidazole ring that exerts physiological properties [10,11,12,13] including the main mechanism responsible for improving physical performance: a high proton buffer capacity [14,15] initiated in the lower physiological range which precedes other intramuscular buffer systems, acting at the earlier stages of exercise [16,17,18]. Compared to other buffer systems, the reported capacity of the non-bicarbonate carnosine system is small [19,20], but the maximum capacity of muscle to store carnosine remains unknown [21], which means that total performance gains by β-alanine supplementation can be greater than what we currently know. Chronic supplementation is conditioned by paresthesia, a harmless sensory side effect of itching [22,23,24,25] that limits the maximum single dose tolerable [26] and therefore the maximum daily dose feasible, consequently prolonging treatment time. Thus, long supplementation studies to observe both its performance benefits and to ascertain carnosine store limits are required [27,28,29,30].

This was mainly solved through a sustained-release tablet that increased the daily dose without enhancing the intensity of paresthesia [31], but to date, no other galenic formulation has been developed to enable larger acute doses which would permit adherence to shorter supplementation protocols with higher daily amounts. Amino acids in sports nutrition supplements are usually in powder form, providing several grams of active ingredients in a single dose, with the practical advantage of avoiding ingestion of several tablets [32,33]. The main problem with β-alanine in pure powder form is that it elicits paresthesia even at low doses due to high peak consequence of its fast absorption [26,31], Thus, a modified-release formulation might represent an alternative to avoid its fast absorption preventing the appearance of the high peak and may allow the administration of larger daily amounts. Galenic modification of powder sport supplements represent a novelty which applies pharmaceutical technology advances to potentially improve some characteristics. According to a mathematical model with slow-release tablets [34], total muscle carnosine increases are (linearly) dependent on daily β-alanine dose, not only to raise daily muscle carnosine synthesis, but also to maintaining high carnosine stores subject to a constant decay. If this outcome is reproduced at higher doses as some evidence suggests [35], higher daily doses can result in either shorter supplementation protocols, or higher carnosine stores over time. Furthermore, recent studies have focused on optimal dosing schedules and individualized pharmacokinetic response using protocols which adjust acute intake based on anthropometric features such as weight [36,37] which requires a β-alanine powder retaining its controlled-release properties (tablet’s sustained-release features may be voided when its physical integrity is compromised—i.e., broken in smaller units [38]). Additionally, the controlled-release powder blend contains l-histidine, which availability in the muscle may be compromised with chronic supplementation [39], and carnosine as an additional donor of both β-alanine and l-histidine after hydrolysis [40,41].

In this study we assess the pharmacokinetics, bioavailability and paresthesia effects of β-alanine at isomolar doses in physically active healthy volunteers after the oral administration of commonly employed slow-release tablets and a novel controlled-release formulation in a crossover study. The aim was to study the pharmacokinetic profile and paresthesia dynamics of a modified-release β-alanine powder as a novel alternative formulation for sport users.

## 2. Methods

### 2.1. Study Design and Participants

A single-blind, cross-over, randomized pharmacokinetic study to compare a novel controlled-release product versus a marketed slow release tablet reference formulation of β-alanine was designed. Participants were a representative sample of the target population of the products and provided written informed consent prior to the commencement of the study. The inclusion criteria were: (a) healthy adults aged 18–40 years old; (b) engaged in physical activity at least three times a week. The exclusion criteria were: (a) current or past history of a serious clinical pathology; (b) supplementation with β-alanine products in the past; (c) hyper-beta-alaninemia; (d) changes to the diet or exercise routine during the study. We recruited a total of twelve subjects.

Dose selection was aimed to provide the highest amount of β-alanine in a single dose without eliciting paresthesia, reducing the number of intakes per day, which would be expected to improve compliance and adherence in chronic supplementation [42]. A previous intravenous infusion study of 9.16 ± 0.78 g at a mean rate of 60.6 ± 5.7 mg/min during 150 min (3.6 g/h and 39.7% of the total amount per hour) of β alanine elicited mild transient paresthesia, which disappeared for all subjects after the infusion was completed [43]. Therefore, we should aim to keep below this limit. A previous in vitro release assay test on the test product was performed and showed an estimated release rate of 16.68% per hour during a total of 6 h [44]. Thus, we estimated that an 8 g dose would yield approximately 1.33 g per hour (approximately half the amount compared to the previously mentioned study [43]), which may result in similar or unperceived paresthesia within the expected intersubject variability [24].

Ethics approval and consent to participate: Ethical approval was granted by the institutional research ethics committee of the Catholic University of Murcia (No. CE021902) and was performed in accordance with the ethical standards as laid down in the 1964 Declaration of Helsinki and its later amendments.

### 2.2. Test and Reference Formulation: Composition

Subjects were randomly assigned to a group for the crossover sequence (Epidat 4.2, 2016) and were administered orally β-alanine 8 g, l-histidine 300 mg, carnosine 100 mg as a controlled-release powder blend test formulation (BETAFOR3MAX^®^, Martinez Nieto S.A., Cartagena, Spain) or a slow release tablet reference formulation (Etixx Sports NV, Merelbeke, Belgium). We discarded the pure β-alanine powder as a feasible reference product, as it may elicit unnecessary severe side effects at these doses due to its potential fast absorption [26]. The test product was a controlled-release powder blend formulation, which contained 8 g of β-alanine, 300 mg of l-histidine and 100 mg of carnosine per dose. The modified-release property was obtained by a specific manufacture process not utilized before in the field of sports supplements in powder formulas. l-Histidine was added to help maintain carnosine synthesis in view of chronic β-alanine periods in which l-histidine availability in muscle may be compromised [39]. Carnosine was included as an additional donor of β-alanine and l-histidine after hydrolysis either before reaching the plasma by cytosolic carnosinase (CN2) [45,46] or once in the plasma by serum carnosinase [40,41]. The reference product was a slow release tablet which required 10 tablets to reach 8 g of β-alanine and 20 mg Zinc.

### 2.3. Test and Reference Formulations: β-Alanine Content Verification

Both products were tested to verify their β-alanine content by HLPC with the following procedure: A 4-level calibration curve prepared with pure β-alanine as reference (P01090, Cambridge Commodities Ltd., Ely, UK) with known purity (99.6%, batch number 201704043) at different concentrations (10, 20, 50 and 100 ppm) was performed. Correlation r^2^ > 0.990 was required to validate the calibration curve after regression analysis by the method of least squares. Additionally, verification after quantification of a known quantity prepared in a solution with the reference standard, must be within 90–110% of this quantity. A sample containing one single dose of each product—10 slow-release tablets, and the amount containing 8 g β-alanine of the controlled-release powder—were weighed in a precision weight scale (Mettler Toledo AT261, Marshall Scientific LLC, Hampton, NH, USA) for content quantification. Samples were diluted in deionized water and derivatized after mixing with reagents for 15 min at 40 °C, further buffered with phosphate. Afterwards, 10 μL of sample was analyzed by HPLC in a Agilent Technology 1200 series system with a 5 µm, 4.6 × 150 mm C18 column (Zorbax Eclipse Plus, Agilent Technologies Inc., Marshfield, MA, USA) and a UV-VIS detector set at 360 nm, flow rate 1 mL/min at 20 °C with a mobile phase of phosphate buffer (pH = 6) and acetonitrile using a gradient elution (t, %B: (0; 10), (10; 25), (15; 50), (19; 50), (20; 10) and (26; 10)). The assay complied with the validation criteria of the Association of Official Analytical Chemists (AOAC), Standard Method Performance Requirements (SMPRs) for Identification and Quantitation of Free Alpha Amino Acids in Dietary Ingredients and Supplements [47].

### 2.4. Study Procedures, Sample Collection and Processing

The reference and the test formulations were provided in a container without any labeling. Subjects were isolated from researchers and participants to avoid interference in their answers. Furthermore, to preserve investigator’s blinding during the trial as well as the posterior data handling, a staff member not involved in the research activities checked that the product was consumed and that the questionnaires were completed. The products were administered in a single oral dose after overnight fasting and water was allowed ad libitum. Five hours into the study, a standardized vegan meal free from β-alanine and l-histidine foods were provided. It included fresh salads, grilled vegetables (cooked and dressed with vegetable oils), egg-free mayonnaise, dried spices and fruit. Subjects were prohibited any different food or drink except water. A 1-week washout period was kept between the two crossover phases.

Blood samples (10 mL) were collected from the antecubital vein at 0 (pre-dose baseline), 30, 60, 90, 120, 150, 180, 210, 240, 300, 360, 420 and 480 min in tripotassium EDTA tubes, centrifuged (4500 rpm, 5 min, 4 °C) the supernatant separated, kept frozen at −20 °C until β-alanine determination by LC-MS/MS liquid chromatography-tandem mass spectrometry (Clinical Analysis Laboratory, Centro medico Vírgen de la Caridad, Cartagena, Spain).

### 2.5. Paresthesia Questionnaires

Paresthesia was assessed concomitantly after each blood sample collection using perceptual ratings questionnaires [31] presented always in the same sequence without allowing the subjects to browse back for revision. Paresthesia intensity was assessed with two scale types. The Visual Analogue Score (VAS) consists of a horizontal, continuous 10 cm line with ending vertical marks labeled “no unusual sensation” and “most intense sensation imaginable”. The subject draws a mark at the distance most fitting with their perception intensity and the output is the segment length from the low end. The Intensity of Sensation Score (ISS) features a discrete category-ratio scale which allows non-linear intensity perception changes with 9 categories ranged from “absent” (1) to “unbearable” (9) with in-between levels: low (2–3), moderate (4–6) and intense (7–8). The subject would tick the best fitting category. Lastly, the subjects were interviewed to identify additional adverse effects.

### 2.6. Pharmacokinetic Data Analysis

Individual non-compartmental pharmacokinetic parameters were calculated using Phoenix^®^ 8.2 (Certara USA, Inc., Princeton, NJ, USA). The maximum β-alanine plasma concentration (C_MAX_) and time to C_MAX_ (T_MAX_) were obtained from the pharmacokinetic profiles; the elimination rate constant (k_el_) was calculated by log-linear regression of the concentration–time data in the terminal slope; the elimination half-life (t_1/2_) was ln2/kel; the area under the curve up to the last concentration (AUC_0__→last_) was calculated using the method of the trapezoids, the extrapolated AUC (AUC_last__→__∞_) was C_last_/k_el_ (C_last_ is the last concentration) and total exposure (AUC_0__→__∞_) was the addition of both areas. The mean residence time MRT_0__→__last_ and MRT_0__→__∞_ were calculated as AUMC_0__→__last_/AUC_0__→__last_ and AUMC_0__→__∞_/AUC_0__→__∞_, respectively, where AUMC is the area under the first moment of the curve. Oral clearance (CL/F) was calculated as D/AUC_0__→__∞_ where D is the dose and F is the bioavailability; the apparent volume of distribution at steady-state (V_SS_/F) was MRT × CL/F. Relative bioavailability was the test to reference AUC_0__→__∞_ ratio. The absorption rate constant Ka was calculated with the equation MRT = MAT + MIT, where MAT is the mean absorption time or 1/K_a_ and MIT is the mean input time or 1/k_el_, assuming no change of k_el_ between oral and intravenous routes. Similarly, the maximum effect (E_MAX_), time to E_MAX_ (TE_MAX_), and area under the effect curve (AUEC) were calculated from the response–time curve.

Pharmacokinetic parameters comparison, crossover sequence and period effects evaluation between the test and the reference outcomes were analyzed using the Wilcoxon test or *t*-test checking for equality of the variances (Phoenix 8.2). In addition, power calculation was performed for all comparisons of key pharmacokinetic parameters between both arms of the study (AUC, Cmax, V/F and Cl/F). They are reported together with the *p*-values.

## 3. Results

Twelve subjects (50% female) were recruited and eleven completed the study (a male subject did not show up on the second occasion). The subjects were 25.6 ± 3.2 years old (range: 24–32 y), weighted 68.1 ± 8.0 kg (50.3–83 kg), had a height of 1.74 ± 0.10 m (1.51–1.83 m) height and a lean mass of 52.1 ± 9.0 kg (38.1–67.6 kg). No side effects were reported except the expected paresthesia.

### 3.1. Pharmacokinetic Analysis

**Figure 1:**
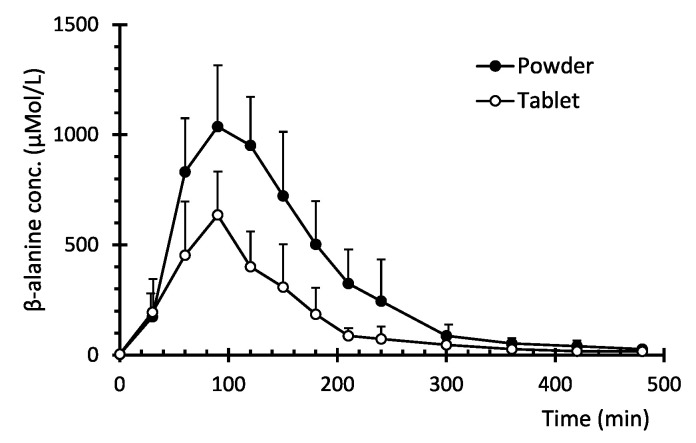
Plasma pharmacokinetic profile of β-alanine after the oral administration of 89.7 mMol to healthy volunteers. (●) controlled-release powder blend formulation, (○) sustained-release tablet. Symbols represents Mean ± SD.

**Figure 2:**
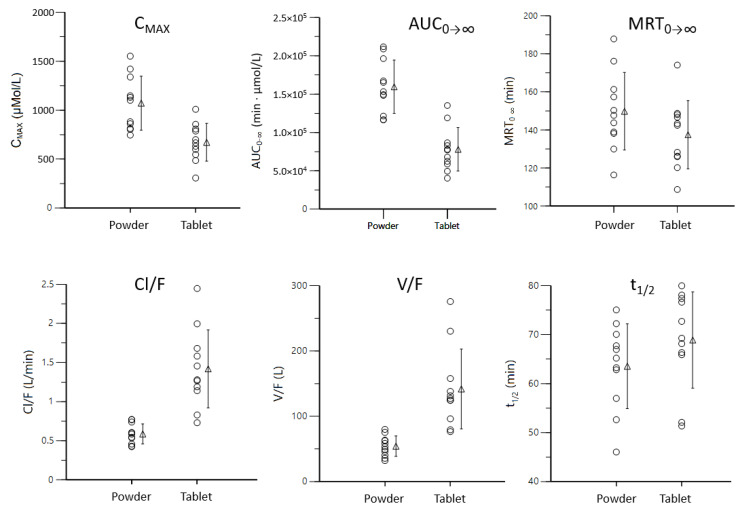
Individual distribution (○) of the main pharmacokinetic parameters obtained by non-compartmental techniques and their mean (∆) and SD.

The plasma concentration–time profiles of β-alanine after administration of either the sustained-release tablet or the controlled-release powder blend are shown in Figure 1, and their non-compartmental pharmacokinetic parameters listed in Table 1 and shown in Figure 2. Statistical differences were found in C_MAX_, AUC, V/F, CL/F and MRT. The C_MAX_, AUC_0__→__last_ and AUC_0__→__∞_ were 1.6-, 2.1- and 2.1-fold higher in the test product and their extrapolated AUC were 1.7% and 2.2%, respectively. This yielded a 2.1-fold β-alanine bioavailability increase of the novel formulation. The K_a_ was 1.5-fold higher in the sustained-release tablet but did not reach statistical differences, and V/F and CL/F decreased 0.38 and 0.41-times in the controlled-release powder blend, parallel to the higher F. Lastly, the MRT_0__→e_ and the MRT_0__→__∞_ were 12% and 8% higher, respectively, in the test formulation, although only the MRT_0__→last_ was statistically significant (*p* = 0.0449).

### 3.2. Pharmacodynamic Analysis

**Figure 3:**
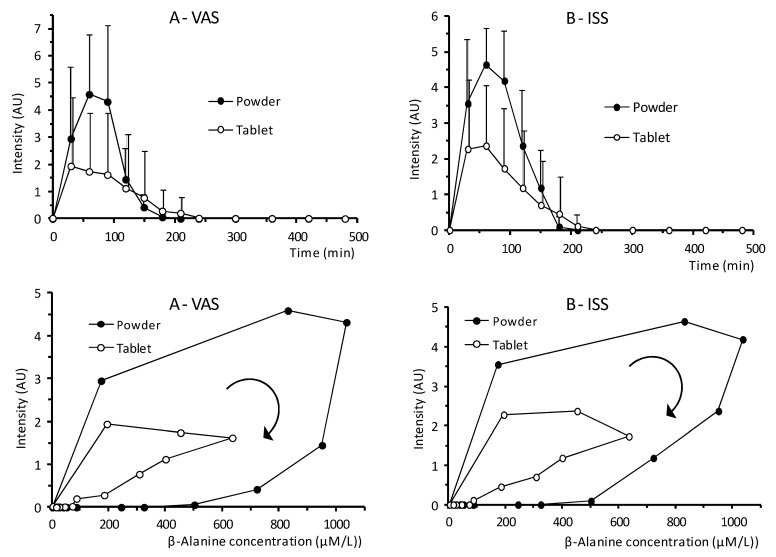
Mean effect intensity time profile (upper panels) and hysteresis plots (lower panels) obtained with the Visual Analog Score (**A**) and the Intensity of Sensation Score (**B**) scales for both formulations. Error bars in the hysteresis plots have been omitted for clarity. Figures in the upper panel represents the mean and positive SD.

Because paresthesia is the limiting side effect of β-alanine dosing schemes, we evaluated it with VAS and the ISS scales. The intensity time course after the administration of each formulation is shown in Figure 3 and the pharmacodynamic parameters listed in Table 2. With the VAS scale, E_MAX_ was 4.95 ± 2.32 AU (arbitrary units) and 2.94 ± 2.90 AU in the test versus the reference product, respectively (*p* = 0.0864). The AUEC was 1.9-fold higher with the test product (not significant). The ISS scale identified statistical differences between both formulations: E_MAX_ and AUEC were 1.7 and 1.9-fold greater in the test product, respectively, and displayed lower variability (Table 2).

## 4. Discussion

### 4.1. Increased Bioavailability

The main finding of the study is the 2.1-fold β-alanine bioavailability (F) increase as well as a 1.9-fold higher C_MAX_ provided by the controlled-release powder blend versus the reference formulation (Figure 1). In addition, the C_MAX_ obtained in the present study is similar to that obtained after oral administration of pure powder at 40 mg/kg (~3.2 g for subjects weighing 80.2 *±* 17.1 kg) [26] and larger AUC (3.3-fold higher) while the dose was ~2.5-fold higher (8 g). This could suggest a more efficient β-alanine absorption after delivery from the controlled-release powder blend.

It is not likely that this higher relative bioavailability (F) is the consequence of lower clearance: first, the elimination rate constant (k_el_) and mean residence time (MRT) remain similar in both formulations, indicating no effect on the disposition process (Table 1); second, the oral clearance (Cl/F) and volume of distribution (V/F) decreased equally, suggesting that only the bioavailability parameter (F) was affected, suggesting that V and Cl may remain unchanged regardless of the formulation. This suggests that the improved bioavailability resulted from an improved absorption of the controlled-release powder compared to the sustained-release tablet.

Therefore, a possible explanation is an improved β-alanine absorption across the gastrointestinal tract, probably involving some of the amino acid absorption mechanisms. Amino acids cannot passively diffuse across membranes but require amino acid transporters (AAT), mostly solute-linked carriers (SLC) transporters. These AATs may modulate their intestinal uptake, renal reabsorption and secure intracellular uptake against a 1000-fold concentration gradient [48]. Experiments carried out in situ in rat small intestine showed pH and Na^+^ dependent [49] uptake and saturable kinetics [50] and studies carried out in brush border membrane vesicles (rabbit) suggested the need of multiple Na^+^ and at least a Cl^−^ associated with β-alanine uptake [51]. Furthermore, characterization in human intestinal caco-2 cells suggested a pH only dependent amino acid transporter at the apical membrane of the enterocytes in the ileum [52,53] and cloning techniques allowed the molecular identification of various ATT, including the SLC6A14 transporter [54]. This transporter is also called the β-alanine carrier, and its small intestine expression is limited to the ileum [55,56]. Functionally, the presence of the β-alanine carrier with saturable absorption kinetics together with their ileum localization would prevent sharp increases of β-alanine ingested in the diet avoiding potential paresthesia. To some extent, the behavior of the controlled-release powder blend may mimic the absorption profile of dietary β-alanine, which ultimately provides a prolonged β-alanine time-course appearance in blood [26,57]. Modified-release devices retarding ability would depend on their physic-chemical properties configuring drug diffusivity [58] and release pattern [59,60]. In this context, the distal intestinal localization may favor continuing absorption from a controlled-release powder formulation contrary to a sustained release tablet that could result in greater unabsorbed dose loss due to the saturable uptake kinetics.

An additional possible explanation of the increased bioavailability may be related to inhibition of β-alanine first-pass metabolism due to the coadministration with l-histidine. A hepatic uptake carrier for neutral and cationic amino acid (ATA3) can be inhibited by several amino acids including β-alanine and histidine with 2-fold greater affinity for histidine than β-alanine [61]. Additionally, β-alanine presents weak inhibition potency to prevent histidine SNAT3 mediated cell uptake [62]. After the coadministration of histidine and β-alanine, histidine may be preferentially uptaken by the hepatic cells instead of β-alanine, thus histidine may play an essential role to diminish the hepatic first-pass uptake of β-alanine resulting in greater circulating levels of β-alanine. Although the role of drug-drug interactions is not fully understood, coadministration has proven a valid strategy to increase bioavailability and modulate tissue uptake [63,64,65,66,67] with potential therapeutic benefits [68,69,70]. If this mechanism were confirmed in future studies, l-histidine addition to β-alanine formulations may play and essential role to improve not only its gastrointestinal absorption but also β-alanine tissue uptake to ensure its muscle [39] and plasma [35,39] concentration after chronic supplementation.

As a limitation of the study, we did not measure β-alanine urinary excretion as our main objective was its bioavailability. Previous studies [26,31,71,72] have shown β-alanine excreted unchanged in urine (~5%). However, a small percentage lost in urine would not be equivalent to a greater uptake by target cells, as it could be eliminated through metabolic routes as previously shown [71,72,73]. However, it remains unclear whether the 2.1-fold higher relative bioavailability would lead to its greater skeletal muscle fibers uptake and further synthesis to carnosine. As transporters, TauT and PAT1 are expressed in human muscle fibers [74]. Whereas TauT is a high-specificity, low capacity β-alanine transporter (which needs to be coupled with a Na and Cl ions) [75] and likely saturated at high concentration of systemic β-alanine, PAT1, on the contrary, is a low-specificity, high-capacity transporter probably less saturable. Although some studies have suggested that TauT plays a dominant role in β-alanine muscle cell uptake, it has also been suggested the existence of additional uptake mechanisms: after intravenous infusion of β-alanine at saturable level, exceeding the maximum transport capacity of TauT in humans a 2.5–3-fold increase in muscle β-alanine was achieved compared to a 1.5-fold increase when sub-saturating amounts were administered [43]. This would support the idea that higher concentrations would achieve greater muscle uptake, even beyond the transport capacity of TauT. However, definitive answer to this hypothesis would require measurements of β-alanine in the biophase, which is beyond the scope of this study.

### 4.2. Paresthesia-Response Analysis

**Figure 4:**
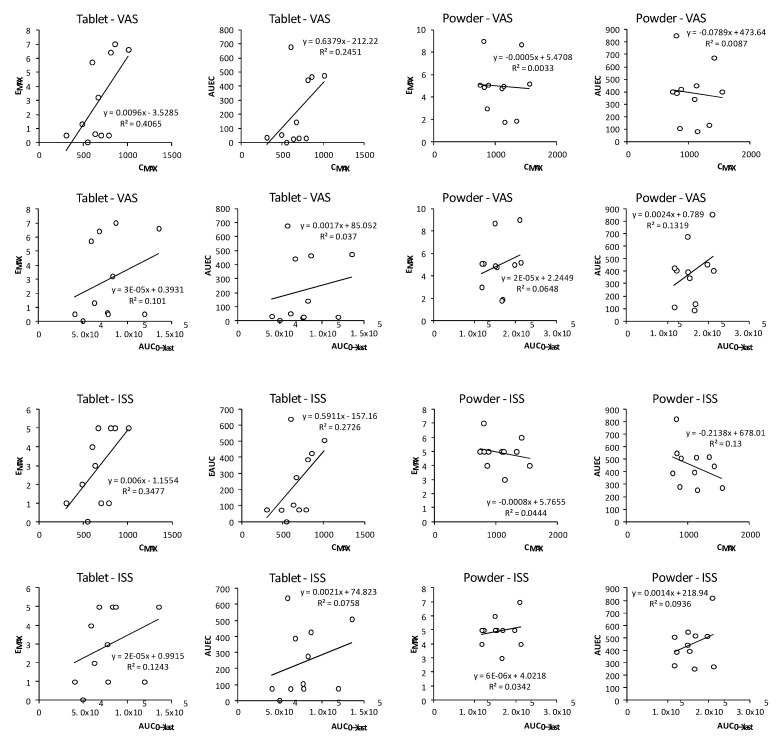
Correlation analysis between the pharmacokinetic parameters C_MAX_, AUC_0__→last_ and the pharmacodynamic parameters E_MAX_ and AUEC obtained with the VAS and the ISS scale for the controlled-released powder blend and sustained-release tablet formulations.

The time course of the paresthesia intensity (Figure 3) shows that the maximum effect, E_MAX_ in the VAS scale was observed earlier than the β-alanine plasma T_MAX_ in the reference and the test formulations (*p* = 0.011 and *p* = 0.006, respectively) and was similar with the ISS scale findings (*p* < 0.001 for both products). This shows that the E_MAX_ is achieved earlier than the plasma C_MAX_ implying saturation of receptors or tolerance, which is consistent with previous reports comparing paresthesia in slow-release tablets and pure powder [31]. This suggests that although the paresthesia effect is dependent on the dose it seems to reach a maximum effect within each formulation, suggesting a non-linear response. Furthermore, we investigated whether C_MAX_ and AUC_0__→__∞_ could predict the paresthesia intensity (E_MAX_, AUEC). However, no correlations were found with any of these pharmacokinetic parameters (Figure 4) with neither formulation. Thus, in the present study we did not find a good predictor of paresthesia intensity and no prediction ability could be identified. This lack of prediction ability would be consistent with saturable kinetics of β-alanine uptake. This would mean that neither C_MAX_ nor total β-alanine systemic exposure serve as consistent predictors of paresthesia intensity. However, this prediction ability remains controversial, as higher pins and needles intensity correlated with higher C_MAX_ after slow-release tablets [31], supporting the role of high C_MAX_ of β-alanine [26]. However, one study showed that subjects that reported paresthesia and other side effects did not have different plasma β-alanine concentration from those not reporting it [41] suggesting a large variability in the response. Likewise. Another study showed that paresthesia intensity was already decreasing albeit higher concentrations (~4-fold) were reached at later time points after intravenous infusion [43]. This behavior is similar to the one found in the current study and supports the findings that paresthesia intensity is not well correlated with plasma peak concentration, it is highly variable and tolerance may be developed. Furthermore, in this study we only tested one dose level; accordingly, no conclusion can be drawn regarding whether the lack of correlation is also due to poor plasma exposure–dose correlation. To address this issue, a pharmacokinetics linearity study would be required.

Both E_MAX_ and AUEC were higher with the controlled-release powder blend (Table 2): E_MAX_ was 1.7-times higher in both scales and AUEC increased 1.8-fold and 1.9-fold using the VAS and the ISS scales, respectively, but only reached statistical difference with the ISS scale. Although bioavailability increased 2.1 times, this raise in paresthesia effect was not attained with the test formulation which further supports the finding that paresthesia intensity is not dose-dependent. Differences between scales may portray their intrinsic differences and the subjective perception of intensity: the VAS is continuous while the ISS is a discrete category-ratio score which excerpt some influence on the subject to decide whether a threshold has been reached to select a specific level in scale [76] (Table 2).

The β-alanine plasma concentration–effect response curves were analyzed to identify possible hysteresis phenomena in the link between effect and pharmacokinetics. This hysteresis plots may present a counter-clockwise or a clockwise loop [77]. A counter-clockwise loop shows increasing effect and may indicate non-instantaneous drug distribution and delay of the response until the drug reaches the biophase. It may also suggest active metabolites or sensitization of the receptors. However, a clockwise loop indicates the highest effect occurs at the beginning and then declines due to tolerance, tachyphylaxis or antagonist active metabolites [77,78]. Our results show a clockwise hysteresis loop (Figure 3) with both scales and formulations, which to the best of our knowledge, has not been described previously. The clockwise loop may indicate a delay in the β-alanine plasma concentration measured in the venous blood (forearm) versus de biophase [78] and could suggest that the receptors in the skin mediating paresthesia [22,23,25] might present a higher affinity for plasma β-alanine even if they may require a minimum threshold to increase intensity to perceived levels. This possibility would be supported by the earlier TE_MAX_ found in both formulations. A change in the sensitivity of the receptors may lead to tolerance [77] or tachyphylaxis when it takes place almost immediately as in our study (Figure 3). This is in line with a case study providing β-alanine intravenously at a high dose (11 g) in which paresthesia decreased and disappeared before the infusion was even completed [79]. In addition, E_MAX_ is reached at the first concentration measured, then it plateaus until the C_MAX_ and returns to the starting point with the tablet formulation. However, with the controlled-release formulation, it takes longer to reach the E_MAX_ which is followed by the decline without the plateau (Figure 3). In fact, with the same plasma concentrations, the effect is greater with the controlled-release powder blend suggesting that saturation of receptors is unlikely. Again, this supports the idea that peak plasma concentration may not be an accurate predictor of paresthesia intensity, challenging previous interpretations and suggesting that other underlying mechanisms can play a more dominant role. As an example, inter-individual characteristics as race (Caucasian or Asian) and sex have been showed to affect timing, location and paresthesia intensity [24]. In the current study, the majority of subjects reported paresthesia and was higher in the controlled-release powder group. To this respect, albeit we may have developed some mechanistic insights, further studies would be needed to assess linearity and dose proportionality, thus we could not predict the maximum dose that would not trigger paresthesia. However, we have shown the possible presence of tolerance and large intersubject variability response.

### 4.3. Implications of the Findings

More efficient release kinetics has been achieved with an innovative controlled-release powder blend delivering 2-fold bioavailability increase of β-alanine after an 8 g oral dose. To our best knowledge, this dose is the highest single acute dose reported in the literature in a single administration, greater than the common daily dose of 6.4 g, and seems more attractive than large number of tablets per intake (10 in this study), or very frequent pure powder quantities split throughout the day. Our results call for researching daily dosing schedules towards new supplementation protocols and the assessment of carnosine synthesis rate with shorter supplementation time. Although formulation effect on carnosine uptake remains unclear [80,81], the ceiling effect seems still far to achieve, being the cumulative dose the essential factor [1,82]. Based on a Bayesian mathematical model [52], the amount of muscle carnosine that may be increased by β-alanine supplementation has not been reached with the usual supplementation protocols (6.4 g/day, 4 weeks) rather it would require around 33 weeks (8 months). In fact, the largest study attempted 24 weeks without evidence of a ceiling effect [21]. Further research to confirm the enhancement capacity of carnosine stores by β-alanine supplementation warrants the development of novel formulations like the controlled-release powder blend used in this study. Achieving higher daily intakes may help to clarify this limit and may allow shorter supplementation strategies. However, we unequivocally cannot conclude out of the present study that these higher doses with an increased uptake may present an advantage over other supplementation strategies. In fact, it is unclear whether high doses eliciting intense but tolerable paresthesia can represent on optimal strategy to raise carnosine synthesis. Overall, data suggests that chronic β-alanine dosing is safe, as β-alanine is an endogenous compound and exhibits a safe profile [83]; however, administration of a larger total cumulative dose, which seems to be unavoidable given the low rate of carnosine synthesis, may require further assessment.

Last, β-alanine controlled-release powder-like delivery systems would avoid the limitations of multiple layer tablets and would allow dosing based on anthropometrical variables or other factors (e.g., body weight, lean muscular mass, etc.) instead of fixed doses [36]. This would lead to assess better the individual responses and to identify new β-alanine supplementation strategies able to reach the minimum cumulative dose to elicit benefits from an earlier stage. Moreover, it may generate a paradigm shift in the usage of β-alanine from chronic to short or mid-term supplementation times in sports nutrition where the expectation of higher performance outcomes in a shorter period of time may overcome daily transient and intense levels of paresthesia.

### 4.4. Future Directions

Assessing the bioavailability of innovative formulations is a necessary step for the development of evidence-based ingredients or blends. Creatine, one of the most studied ergogenic aids in sports nutrition, is marketed as creatine salts associated with different conjugates like creatine citrate, malate and orotate, or as creatine derivates like creatine ethyl-ester [84]. Generally, they have poor bioavailability and not enough active ingredients to reach the bloodstream [85]. In the present study we found that this first step was accomplished with the controlled-release powder blend, ensuring greater systemic exposure after oral administration of the active ingredient than a reference slow-release tablet. This means that for the same amount declared in the label (dose), a greater amount reaches the systemic circulation which in turn could provide a larger physiological effect at the same declared dose. Clearly, the novel powder blend represents a solution to include controlled-release β-alanine to multi-ingredient powder products (the most common format of sports nutrition ergogenic aids) and would enable further research to explore drug-drug interaction in sports nutrition.

## 5. Conclusions

This study shows the suitability of a new controlled-release powder blend formulation to deliver β-alanine systemically orally with a 2.1-fold bioavailability increase versus a slow release tablet formulation. That is to say, twice the amount of β-alanine would reach the systemic circulation when provided as a controlled-release powder blend instead of as a sustained-release tablet. In addition, the 8 g oral dose of β-alanine produced no additional side effects except the expected paresthesia, although its extent would need assessment at high doses chronically administered to ensure a complete safety assessment.

The pharmacokinetic-pharmacodynamic analysis shows a clockwise hysteresis loop with marked tachyphylaxis. Furthermore, no paresthesia prediction between C_MAX_, AUC_0__→__∞_ and E_MAX_, AUEC was found probably due diverse uptake kinetics and effect mechanisms involved, which suggest that peak concentrations are not a good predictor of paresthesia intensity.

This novel controlled-release formulation invites to explore a change from chronic to short or mid-term β-alanine supplementation strategies to enhance carnosine uptake with precision dose calculation considering anthropometrical variables and preserving its controlled-release features.

## Figures and Tables

**Table 1:** Non-compartmental pharmacokinetic parameters of the test and reference formulations.

Parameter (Units)	Powder Blend	Tablet	*p*-Value (Power)
k_e_ (1/min)	0.0111 ± 0.002	0.0103 ± 0.0020	0.2850 (0.19)
t_1/2_ (min)	63.5 ± 8.7	68.9 ± 9.8	0.1994 (0.27)
T_MAX_ (min)	90 ± 13	82 ± 14	0.2767 (0.28)
K_a_ (min^−1^)	0.0199 ± 0.0107	0.0299 ± 0.0121	0.0834 (0.49)
C_MAX_ (µmol/L)	1072 ± 276	672 ± 192	0.0045 (0.86)
AUC_0__→last_ (min∙µmol/L)	157,058 ± 34,097	76,314 ± 27,838	<0.0001 (0.96)
AUC_0__→__∞_ (min∙µmol/L)	159,557 ± 34,708	77,965 ± 28,275	<0.0001 (0.96)
V/F (L)	54.2 ± 15.5	142.0 ± 60.9	0.0004 (0.91)
Cl/F (L/min)	0.587 ± 0.128	1.417 ± 0.498	<0.0001 (0.94)
MRT_0__→last_ (min)	143 ± 19	128 ± 16	0.0449 (0.51)
MRT_0__→__∞_ (min)	150 ± 20	138 ± 18	0.1373 (0.31)

Data are presented as mean ± SD. *n* = 11, statistical differences were accepted when *p* < 0.05.

**Table 2:** Model independent analysis of the paresthesia effect of β-alanine in a powder blend or tablet.

Test	Parameter	Powder Blend	Tablet	*p-*Value (Power)
Visual Analogue Score	TE_MAX_ (min)	65.5 ± 22.5 (34.4%)	49.1 ± 33.6 (68.4%)	0.195 (0.26)
	E_MAX_ (AU)	4.95 ± 2.32 (46.7%)	2.94 ± 2.90 (98.8%)	0.086 (0.40)
	AUEC_0__→last_ (AU)	389.1 ± 232.6 (59.8%)	216.4 ± 247.4 (114.5%)	0.107 (0.40)
Intensity of Sensation Score	TE_MAX_ (min)	57.3 ± 21.0 (36.7%)	38.2 ± 23.6 (61.8%)	0.059 (0.47)
	E_MAX_ (AU)	4.91 ± 1.04 (21.3%)	2.91 ± 1.97 (67.8%)	0.009 (0.72)
	AUEC_0__→last_ (AU)	448.8 ± 163.8 (36.4%)	240.1 ± 217.7 (90.7%)	0.019 (0.62)

Data are presented as mean ± SD, coefficient of variation, (CV). AU: arbitrary units unless stated, statistical differences were accepted when *p* < 0.05. Dose administered was of 89.7 mMol in each case.

## Data Availability

The datasets that supports the central findings of this study are presented within the article. Additional data are available on request from the corresponding author.
